# Methyl 2-{1-[(*Z*)-3-methyl-5-oxo-1-phenyl-4,5-dihydro-1*H*-pyrazol-4-yl­idene]ethyl­amino}-3-phenyl­propanoate

**DOI:** 10.1107/S1600536810017241

**Published:** 2010-05-15

**Authors:** Hualing Zhu, Jun Shi, Zhen Wei, Ronghua Dai, Xin Zhang

**Affiliations:** a Department of Basic Science, Tianjin Agriculturial College, Tianjin Jinjing Road No. 22, Tianjin 300384, People’s Republic of China; b Department of Chemistry and Life Science, Tianjin Normal University, Tianjin 300387, People’s Republic of China

## Abstract

The mol­ecule of the title compound, C_22_H_23_N_3_O_3_, exists in the enamine–keto form. A strong intra­molecular N—H⋯O hydrogen bond occurs, generating an *S*(6) ring. The dihedral angle between the heterocycle and the adjacent phenyl ring is 3.75 (15)°.

## Related literature

For the anti­bacterial activity of Schiff bases derived from 4-acyl-5-pyrazolones and metal complexes, see: Li *et al.* (1997 ▶, 2004 ▶). For the biological activity of amino acid esters, see: Xiong *et al.* (1993 ▶). For related structures, see: Wang *et al.* (2003 ▶); Zhang *et al.* (2004 ▶, 2010 ▶); Zhu *et al.* (2005 ▶).
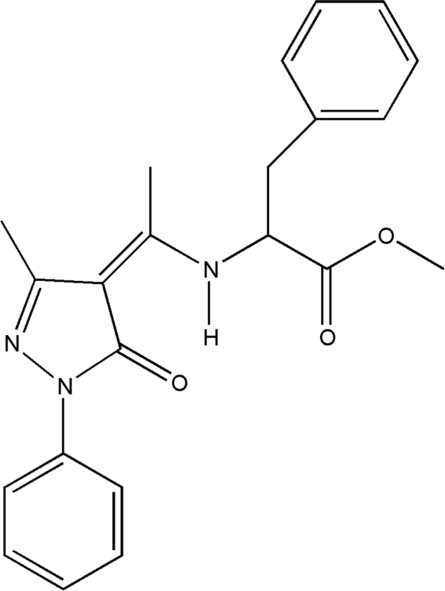


## Experimental

### 

#### Crystal data

C_22_H_23_N_3_O_3_
*M* *_r_* = 377.43
Monoclinic, 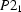

*a* = 10.940 (1) Å
*b* = 7.2105 (7) Å
*c* = 12.867 (1) Å
β = 92.718 (2)°
*V* = 1013.84 (16) Å^3^
*Z* = 2
Mo *K*α radiation
μ = 0.08 mm^−1^
*T* = 296 K
0.28 × 0.12 × 0.10 mm

#### Data collection

Bruker SMART CCD area-detector diffractometer
Absorption correction: multi-scan (*SADABS*; Sheldrick, 2000\bbr00) *T*_min_ = 0.977, *T*_max_ = 0.988
6036 measured reflections
2366 independent reflections
1239 reflections with *I* > 2σ(*I*)
*R*_int_ = 0.028

#### Refinement

*R*[*F* ^2^ > 2σ(*F* ^2^)] = 0.037
*wR*(*F* ^2^) = 0.096
*S* = 1.01
2366 reflections
256 parameters
1 restraint
H-atom parameters constrained
Δρ_max_ = 0.08 e Å^−3^
Δρ_min_ = −0.16 e Å^−3^

### 

Data collection: *SMART* (Bruker, 1999 ▶); cell refinement: *SAINT* (Bruker, 1999 ▶); data reduction: *SAINT*; program(s) used to solve structure: *SHELXS97* (Sheldrick, 2008 ▶); program(s) used to refine structure: *SHELXL97* (Sheldrick, 2008 ▶); molecular graphics: *ORTEP-3* (Farrugia, 1997 ▶); software used to prepare material for publication: *SHELXL97*.

## Supplementary Material

Crystal structure: contains datablocks I, global. DOI: 10.1107/S1600536810017241/lx2128sup1.cif

Structure factors: contains datablocks I. DOI: 10.1107/S1600536810017241/lx2128Isup2.hkl

Additional supplementary materials:  crystallographic information; 3D view; checkCIF report

## Figures and Tables

**Table 1:** Hydrogen-bond geometry (Å, °)

*D*—H⋯*A*	*D*—H	H⋯*A*	*D*⋯*A*	*D*—H⋯*A*
N3—H3⋯O1	0.86	1.98	2.695 (3)	141

**Fig. 1.**
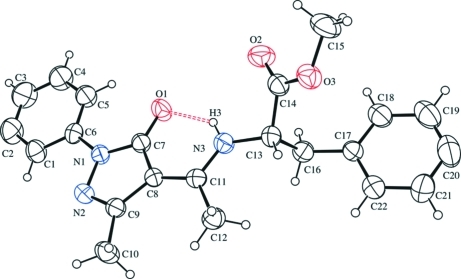
The molecular structure of the title compound with the atom numbering scheme. Displacement ellipsoids are drawn at the 30% probability level. H atoms are presented as a small spheres of arbitrary radius.

## supplementary crystallographic information

### Comment

The Schiff bases derived from 4-acyl-5-pyrazolones and their metal complexes have been studied widely for their high antibacterial activation [Li *et al.*, 1997, 2004]. Since amino acid esters also possess good antibacterial and biological activations (Xiong *et al.*, 1993), several Structures of Schiff bases derived from 4-acyl-5-pyrazolones and amino acid esters and closely related to the title compound have been reported [Zhu *et al.*, 2005; Zhang *et al.*, 2010]. We report the the crystal structure of the title compound.

In the molecule of the title compound, (Fig.1) atoms O1, C7, C8, C11 and atom N3 form a plane, the largest deviation being 0.024 (2) Å for atom C11. The dihedral angle between this mean plane and the pyrazolone ring of PMAP is 1.44 (3)°, indicating that they are essentially coplanar, as seen in Ethyl 2-{[(1*Z*)-(3- methyl-5-oxo-1-phenyl-4,5-dihydro-1*H*-pyrazol-4-ylidene) (*p*-tolyl)methyl]amino}-3-phenylpropanoate (1.52 (4)°; Zhang *et al.*, 2010). The bond lengths within this part of the molecular lie between classical single-and double-bond lengths, indicating extensive conjugation. Atoms N3, C13, C14 and O2 are not coplanar, the torsion angle is -39.0 (4)°, similar to some other 4-acylpyrazolone schiff bases (Zhang *et al.* 2004; Wang *et al.* 2003). The bond lengths in this part of the molecular indicate that only C14—O2 is classical double bond, other bonds are classical single bonds. A strong intramolecular hydrogen bond N3—H3···O1 is observed (Table 1 & Fig. 1), stabilizing to an enamine–keto form.

### Experimental

The title compound was synthesized by refluxing the mixture of HPMAP (15 m mol) and phenylalanine methyl ester (15*m* mol) in ethanol (100 ml) over a steam bath for about 4 h, then the solution was cooled down to room temperature. After four days, white block was obtained and dried in air. The product was recrystallized from ethanol which afforded colorless and acerate crystals suitable for X-ray analysis.

### Refinement

In the absence of significant anomalous scattering effect, 1127 Friedel pairs were merged. All H atoms were geometrically positioned and refined using a riding model, with C—H = 0.93 Å for the aryl, 0.97 Å for methylene, 0.98 Å for methyne and 0.96 Å for the methyl H atoms. *U*_iso_(H)= 1.2 *U*_eq_(C) for aryl, methylene and methyne, and 1.5*U*_eq_(C) for methyl H atoms.

### Crystal data

**Table:**

C_22_H_23_N_3_O_3_	*F*(000) = 400
*M**_r_* = 377.43	*D*_x_ = 1.236 Mg m^−^^3^
Monoclinic, *P*2_1_	Mo *K*α radiation, λ = 0.71073 Å
*a* = 10.940 (1) Å	Cell parameters from 1220 reflections
*b* = 7.2105 (7) Å	θ = 3.2–22.4°
*c* = 12.867 (1) Å	µ = 0.08 mm^−^^1^
β = 92.718 (2)°	*T* = 296 K
*V* = 1013.84 (16) Å^3^	Block, colorless
*Z* = 2	0.28 × 0.12 × 0.10 mm

### Data collection

**Table:**

Bruker SMART CCD area-detector diffractometer	2366 independent reflections
Radiation source: fine-focus sealed tube	1239 reflections with *I* > 2σ(*I*)
graphite	*R*_int_ = 0.028
Detector resolution: 10.0 pixels mm^-1^	θ_max_ = 27.0°, θ_min_ = 1.6°
φ and ω scans	*h* = −13→11
Absorption correction: multi-scan (*SADABS*; Sheldrick, 2000\bbr00)	*k* = −9→9
*T*_min_ = 0.977, *T*_max_ = 0.988	*l* = −13→16
6036 measured reflections	

### Refinement

**Table:**

Refinement on *F*^2^	Primary atom site location: structure-invariant direct methods
Least-squares matrix: full	Secondary atom site location: difference Fourier map
*R*[*F*^2^ > 2σ(*F*^2^)] = 0.037	Hydrogen site location: difference Fourier map
*wR*(*F*^2^) = 0.096	H-atom parameters constrained
*S* = 1.01	*w* = 1/[σ^2^(*F*_o_^2^) + (0.0409*P*)^2^] where *P* = (*F*_o_^2^ + 2*F*_c_^2^)/3
2366 reflections	(Δ/σ)_max_ = 0.001
256 parameters	Δρ_max_ = 0.08 e Å^−^^3^
1 restraint	Δρ_min_ = −0.16 e Å^−^^3^

### Special details

**Table:**

Geometry. All esds (except the esd in the dihedral angle between two l.s. planes) are estimated using the full covariance matrix. The cell esds are taken into account individually in the estimation of esds in distances, angles and torsion angles; correlations between esds in cell parameters are only used when they are defined by crystal symmetry. An approximate (isotropic) treatment of cell esds is used for estimating esds involving l.s. planes.
Refinement. Refinement of F^2^ against ALL reflections. The weighted R-factor wR and goodness of fit S are based on F^2^, conventional R-factors R are based on F, with F set to zero for negative F^2^. The threshold expression of F^2^ > 2sigma(F^2^) is used only for calculating R-factors(gt) etc. and is not relevant to the choice of reflections for refinement. R-factors based on F^2^ are statistically about twice as large as those based on F, and R- factors based on ALL data will be even larger.

### Fractional atomic coordinates and isotropic or equivalent isotropic displacement parameters (Å^2^)

**Table:**

	*x*	*y*	*z*	*U*_iso_*/*U*_eq_	
O1	0.69537 (16)	0.7004 (4)	0.63222 (13)	0.0812 (6)	
O2	0.9385 (2)	0.5317 (4)	0.4573 (2)	0.0951 (8)	
O3	0.9296 (2)	0.4530 (3)	0.28897 (18)	0.0818 (7)	
N1	0.49129 (18)	0.7209 (4)	0.67625 (16)	0.0594 (6)	
N2	0.37536 (19)	0.7237 (4)	0.62394 (18)	0.0662 (6)	
N3	0.7085 (2)	0.6887 (4)	0.42371 (17)	0.0681 (7)	
H3	0.7408	0.6814	0.4858	0.082*	
C1	0.3903 (3)	0.7353 (6)	0.8383 (2)	0.0798 (10)	
H1	0.3156	0.7472	0.8012	0.096*	
C2	0.3948 (4)	0.7310 (7)	0.9457 (3)	0.1043 (12)	
H2	0.3226	0.7392	0.9809	0.125*	
C3	0.5037 (5)	0.7148 (8)	1.0007 (3)	0.1151 (14)	
H3A	0.5060	0.7114	1.0730	0.138*	
C4	0.6103 (3)	0.7035 (8)	0.9487 (2)	0.1075 (13)	
H4	0.6849	0.6941	0.9860	0.129*	
C5	0.6072 (3)	0.7060 (6)	0.8416 (2)	0.0853 (10)	
H5	0.6794	0.6971	0.8066	0.102*	
C6	0.4968 (3)	0.7219 (5)	0.7866 (2)	0.0626 (7)	
C7	0.5836 (2)	0.7091 (5)	0.6073 (2)	0.0608 (7)	
C8	0.5237 (2)	0.7100 (5)	0.5063 (2)	0.0534 (6)	
C9	0.3959 (2)	0.7185 (5)	0.5250 (2)	0.0595 (7)	
C10	0.2866 (2)	0.7155 (6)	0.4494 (2)	0.0834 (10)	
H10A	0.2130	0.7153	0.4871	0.125*	
H10B	0.2892	0.6059	0.4072	0.125*	
H10C	0.2880	0.8233	0.4056	0.125*	
C11	0.5879 (2)	0.7059 (5)	0.4158 (2)	0.0563 (7)	
C12	0.5269 (2)	0.7185 (6)	0.3096 (2)	0.0727 (8)	
H12A	0.5209	0.5969	0.2794	0.109*	
H12B	0.5741	0.7971	0.2666	0.109*	
H12C	0.4463	0.7697	0.3146	0.109*	
C13	0.7929 (2)	0.6804 (5)	0.3398 (2)	0.0646 (8)	
H13	0.7495	0.6375	0.2759	0.077*	
C14	0.8937 (3)	0.5450 (5)	0.3710 (3)	0.0677 (9)	
C15	1.0389 (3)	0.3383 (6)	0.3056 (3)	0.1081 (14)	
H15A	1.0281	0.2559	0.3631	0.162*	
H15B	1.1086	0.4164	0.3206	0.162*	
H15C	1.0518	0.2670	0.2441	0.162*	
C16	0.8488 (3)	0.8723 (5)	0.3216 (3)	0.0724 (9)	
H16A	0.7833	0.9626	0.3131	0.087*	
H16B	0.8991	0.9071	0.3826	0.087*	
C17	0.9252 (3)	0.8793 (4)	0.2280 (3)	0.0645 (8)	
C18	1.0502 (3)	0.8632 (5)	0.2357 (3)	0.0886 (11)	
H18	1.0890	0.8466	0.3009	0.106*	
C19	1.1202 (4)	0.8709 (6)	0.1491 (5)	0.1149 (16)	
H19	1.2049	0.8596	0.1561	0.138*	
C20	1.0639 (5)	0.8953 (7)	0.0535 (4)	0.1186 (16)	
H20	1.1104	0.9008	−0.0051	0.142*	
C21	0.9411 (5)	0.9114 (7)	0.0431 (3)	0.1104 (14)	
H21	0.9030	0.9288	−0.0223	0.133*	
C22	0.8718 (3)	0.9019 (5)	0.1308 (3)	0.0847 (11)	
H22	0.7871	0.9111	0.1231	0.102*	

### Atomic displacement parameters (Å^2^)

**Table:**

	*U*^11^	*U*^22^	*U*^33^	*U*^12^	*U*^13^	*U*^23^
O1	0.0527 (12)	0.1241 (19)	0.0665 (13)	−0.0042 (16)	−0.0012 (9)	0.0002 (17)
O2	0.0909 (17)	0.113 (2)	0.0801 (18)	0.0257 (16)	−0.0114 (13)	0.0023 (16)
O3	0.0824 (16)	0.0755 (15)	0.0886 (19)	0.0130 (13)	0.0146 (13)	−0.0103 (14)
N1	0.0538 (13)	0.0666 (16)	0.0580 (15)	−0.0033 (16)	0.0047 (11)	0.0003 (17)
N2	0.0544 (14)	0.0725 (17)	0.0719 (16)	0.0014 (15)	0.0045 (12)	0.0012 (19)
N3	0.0611 (15)	0.088 (2)	0.0548 (14)	0.0066 (16)	0.0041 (11)	0.0009 (17)
C1	0.077 (2)	0.092 (3)	0.071 (2)	0.000 (2)	0.0145 (16)	−0.010 (2)
C2	0.113 (3)	0.128 (4)	0.075 (3)	0.004 (3)	0.029 (2)	−0.012 (3)
C3	0.138 (3)	0.148 (4)	0.060 (2)	0.007 (4)	0.009 (2)	−0.007 (3)
C4	0.105 (3)	0.150 (4)	0.066 (2)	−0.003 (4)	−0.004 (2)	0.002 (4)
C5	0.076 (2)	0.115 (3)	0.064 (2)	−0.001 (3)	0.0007 (16)	0.001 (3)
C6	0.0731 (19)	0.0571 (18)	0.0586 (19)	−0.0032 (19)	0.0125 (15)	−0.001 (2)
C7	0.0567 (18)	0.0596 (19)	0.0664 (18)	−0.001 (2)	0.0063 (15)	0.001 (2)
C8	0.0504 (15)	0.0552 (17)	0.0546 (16)	−0.0013 (17)	0.0036 (13)	0.001 (2)
C9	0.0558 (17)	0.0545 (17)	0.0681 (19)	−0.0005 (18)	0.0007 (13)	0.002 (2)
C10	0.0553 (17)	0.110 (3)	0.084 (2)	0.002 (2)	−0.0061 (14)	−0.004 (3)
C11	0.0563 (18)	0.0512 (17)	0.0609 (18)	−0.0009 (18)	−0.0023 (13)	0.000 (2)
C12	0.0757 (19)	0.080 (2)	0.0625 (18)	0.009 (2)	0.0011 (14)	0.002 (2)
C13	0.0619 (18)	0.076 (2)	0.0568 (18)	0.0028 (18)	0.0082 (14)	0.0025 (18)
C14	0.057 (2)	0.066 (2)	0.080 (3)	−0.0016 (17)	0.0078 (19)	0.000 (2)
C15	0.091 (3)	0.089 (3)	0.146 (4)	0.037 (2)	0.018 (2)	−0.006 (3)
C16	0.076 (2)	0.069 (2)	0.073 (2)	0.0084 (18)	0.0089 (18)	0.0011 (19)
C17	0.060 (2)	0.064 (2)	0.069 (2)	0.0048 (17)	0.0073 (17)	0.0016 (18)
C18	0.073 (3)	0.092 (3)	0.102 (3)	0.003 (2)	0.008 (2)	0.000 (2)
C19	0.072 (3)	0.116 (4)	0.158 (5)	−0.002 (3)	0.026 (3)	0.001 (4)
C20	0.115 (4)	0.107 (4)	0.139 (5)	0.001 (3)	0.060 (3)	0.007 (3)
C21	0.121 (4)	0.127 (4)	0.086 (3)	0.011 (3)	0.027 (3)	0.014 (3)
C22	0.074 (2)	0.101 (3)	0.079 (3)	0.010 (2)	0.010 (2)	0.004 (2)

### Geometric parameters (Å, °)

**Table:**

O1—C7	1.251 (3)	C10—H10B	0.9600
O2—C14	1.197 (4)	C10—H10C	0.9600
O3—C14	1.322 (4)	C11—C12	1.495 (3)
O3—C15	1.462 (4)	C12—H12A	0.9600
N1—C7	1.378 (3)	C12—H12B	0.9600
N1—N2	1.407 (3)	C12—H12C	0.9600
N1—C6	1.418 (3)	C13—C14	1.512 (4)
N2—C9	1.304 (3)	C13—C16	1.535 (4)
N3—C11	1.325 (3)	C13—H13	0.9800
N3—C13	1.454 (3)	C15—H15A	0.9600
N3—H3	0.8600	C15—H15B	0.9600
C1—C6	1.372 (4)	C15—H15C	0.9600
C1—C2	1.382 (4)	C16—C17	1.499 (4)
C1—H1	0.9300	C16—H16A	0.9700
C2—C3	1.362 (5)	C16—H16B	0.9700
C2—H2	0.9300	C17—C22	1.365 (4)
C3—C4	1.374 (5)	C17—C18	1.371 (4)
C3—H3A	0.9300	C18—C19	1.382 (5)
C4—C5	1.376 (4)	C18—H18	0.9300
C4—H4	0.9300	C19—C20	1.361 (5)
C5—C6	1.376 (4)	C19—H19	0.9300
C5—H5	0.9300	C20—C21	1.349 (5)
C7—C8	1.427 (3)	C20—H20	0.9300
C8—C11	1.388 (3)	C21—C22	1.390 (5)
C8—C9	1.431 (3)	C21—H21	0.9300
C9—C10	1.506 (3)	C22—H22	0.9300
C10—H10A	0.9600		
			
C14—O3—C15	116.0 (3)	C11—C12—H12B	109.5
C7—N1—N2	111.3 (2)	H12A—C12—H12B	109.5
C7—N1—C6	130.4 (2)	C11—C12—H12C	109.5
N2—N1—C6	118.2 (2)	H12A—C12—H12C	109.5
C9—N2—N1	105.9 (2)	H12B—C12—H12C	109.5
C11—N3—C13	127.7 (2)	N3—C13—C14	108.1 (3)
C11—N3—H3	116.1	N3—C13—C16	110.4 (3)
C13—N3—H3	116.1	C14—C13—C16	109.4 (2)
C6—C1—C2	119.5 (3)	N3—C13—H13	109.7
C6—C1—H1	120.2	C14—C13—H13	109.7
C2—C1—H1	120.2	C16—C13—H13	109.7
C3—C2—C1	120.7 (3)	O2—C14—O3	125.2 (3)
C3—C2—H2	119.7	O2—C14—C13	124.0 (3)
C1—C2—H2	119.7	O3—C14—C13	110.7 (3)
C2—C3—C4	119.6 (3)	O3—C15—H15A	109.5
C2—C3—H3A	120.2	O3—C15—H15B	109.5
C4—C3—H3A	120.2	H15A—C15—H15B	109.5
C3—C4—C5	120.4 (3)	O3—C15—H15C	109.5
C3—C4—H4	119.8	H15A—C15—H15C	109.5
C5—C4—H4	119.8	H15B—C15—H15C	109.5
C6—C5—C4	119.7 (3)	C17—C16—C13	113.2 (3)
C6—C5—H5	120.2	C17—C16—H16A	108.9
C4—C5—H5	120.2	C13—C16—H16A	108.9
C1—C6—C5	120.1 (3)	C17—C16—H16B	108.9
C1—C6—N1	119.3 (3)	C13—C16—H16B	108.9
C5—C6—N1	120.6 (3)	H16A—C16—H16B	107.7
O1—C7—N1	125.1 (2)	C22—C17—C18	117.3 (3)
O1—C7—C8	129.4 (2)	C22—C17—C16	120.6 (3)
N1—C7—C8	105.5 (2)	C18—C17—C16	122.1 (3)
C11—C8—C7	122.3 (2)	C17—C18—C19	121.8 (4)
C11—C8—C9	132.8 (2)	C17—C18—H18	119.1
C7—C8—C9	104.9 (2)	C19—C18—H18	119.1
N2—C9—C8	112.4 (2)	C20—C19—C18	119.3 (4)
N2—C9—C10	117.6 (2)	C20—C19—H19	120.3
C8—C9—C10	130.0 (2)	C18—C19—H19	120.3
C9—C10—H10A	109.5	C21—C20—C19	120.5 (4)
C9—C10—H10B	109.5	C21—C20—H20	119.8
H10A—C10—H10B	109.5	C19—C20—H20	119.8
C9—C10—H10C	109.5	C20—C21—C22	119.6 (4)
H10A—C10—H10C	109.5	C20—C21—H21	120.2
H10B—C10—H10C	109.5	C22—C21—H21	120.2
N3—C11—C8	118.6 (2)	C17—C22—C21	121.6 (3)
N3—C11—C12	118.4 (2)	C17—C22—H22	119.2
C8—C11—C12	122.9 (2)	C21—C22—H22	119.2
C11—C12—H12A	109.5		
			
C7—N1—N2—C9	−1.6 (4)	C7—C8—C9—C10	−177.3 (4)
C6—N1—N2—C9	−178.8 (3)	C13—N3—C11—C8	179.6 (3)
C6—C1—C2—C3	0.4 (7)	C13—N3—C11—C12	0.2 (5)
C1—C2—C3—C4	0.3 (9)	C7—C8—C11—N3	4.4 (5)
C2—C3—C4—C5	−0.8 (9)	C9—C8—C11—N3	−176.7 (3)
C3—C4—C5—C6	0.6 (8)	C7—C8—C11—C12	−176.1 (3)
C2—C1—C6—C5	−0.6 (6)	C9—C8—C11—C12	2.7 (6)
C2—C1—C6—N1	178.2 (4)	C11—N3—C13—C14	−143.2 (3)
C4—C5—C6—C1	0.1 (7)	C11—N3—C13—C16	97.2 (4)
C4—C5—C6—N1	−178.7 (4)	C15—O3—C14—O2	−5.2 (5)
C7—N1—C6—C1	179.9 (4)	C15—O3—C14—C13	171.1 (3)
N2—N1—C6—C1	−3.4 (5)	N3—C13—C14—O2	−39.0 (4)
C7—N1—C6—C5	−1.2 (6)	C16—C13—C14—O2	81.2 (4)
N2—N1—C6—C5	175.4 (3)	N3—C13—C14—O3	144.8 (3)
N2—N1—C7—O1	−178.4 (3)	C16—C13—C14—O3	−95.1 (3)
C6—N1—C7—O1	−1.7 (6)	N3—C13—C16—C17	−173.2 (3)
N2—N1—C7—C8	1.8 (4)	C14—C13—C16—C17	68.1 (4)
C6—N1—C7—C8	178.6 (3)	C13—C16—C17—C22	82.0 (4)
O1—C7—C8—C11	−1.9 (6)	C13—C16—C17—C18	−97.8 (4)
N1—C7—C8—C11	177.8 (3)	C22—C17—C18—C19	0.6 (5)
O1—C7—C8—C9	179.0 (4)	C16—C17—C18—C19	−179.6 (4)
N1—C7—C8—C9	−1.3 (4)	C17—C18—C19—C20	0.0 (7)
N1—N2—C9—C8	0.7 (4)	C18—C19—C20—C21	−0.1 (7)
N1—N2—C9—C10	178.6 (3)	C19—C20—C21—C22	−0.4 (7)
C11—C8—C9—N2	−178.6 (4)	C18—C17—C22—C21	−1.1 (6)
C7—C8—C9—N2	0.4 (4)	C16—C17—C22—C21	179.1 (4)
C11—C8—C9—C10	3.8 (7)	C20—C21—C22—C17	1.0 (7)

### Hydrogen-bond geometry (Å, °)

**Table:**

*D*—H···*A*	*D*—H	H···*A*	*D*···*A*	*D*—H···*A*
N3—H3···O1	0.86	1.98	2.695 (3)	141
